# Assessing the Relationship Between Emotional States of Dogs and Their Human Handlers, Using Simultaneous Behavioral and Cardiac Measures

**DOI:** 10.3389/fvets.2022.897287

**Published:** 2022-07-11

**Authors:** Emma K. Grigg, Serene Liu, Denise G. Dempsey, Kylee Wong, Melissa Bain, John J. Sollers, Rani Haddock, Lori R. Kogan, Jennifer A. Barnhard, Ashley A. Tringali, Abigail P. Thigpen, Lynette A. Hart

**Affiliations:** ^1^Department of Population Health and Reproduction, School of Veterinary Medicine, University of California, Davis, Davis, CA, United States; ^2^Graduate Group in Epidemiology, University of California, Davis, Davis, CA, United States; ^3^StressReductionPrograms.com, Davis, CA, United States; ^4^Animal Biology, University of California, Davis, Davis, CA, United States; ^5^Department of Medicine and Epidemiology, School of Veterinary Medicine, University of California, Davis, Davis, CA, United States; ^6^Department of Psychology, North Carolina Central University, Durham, NC, United States; ^7^Clinical Sciences Department, College of Veterinary Medicine and Biomedical Sciences, Colorado State University, Fort Collins, CO, United States; ^8^Bergin University of Canine Studies, Penngrove, CA, United States; ^9^Equine Medical Emergency, Critical Care and Neonatology Service, School of Veterinary Medicine, University of California, Davis, Davis, CA, United States; ^10^Department of Animal Science, University of California, Davis, Davis, CA, United States

**Keywords:** human-animal interactions, domestic dogs, familiarity, veterinary clinic, emotional contagion, low stress handling, heart rate variability, welfare

## Abstract

Negative stress due to human handling has been reported for a number of domestic animals, including dogs. Many companion dogs display significant stress during routine care in the veterinary clinic, risking injury to staff and potentially compromising the quality of care that these dogs receive. On the other hand, positive interactions with humans can have a beneficial effect on dogs, particularly in stressful situations such as animal shelters. Research has shown that dogs can detect human emotions through visual, auditory, and chemical channels, and that dogs will exhibit emotional contagion, particularly with familiar humans. This study investigated relationships between emotional states of dogs and unfamiliar human handlers, using simultaneous measures of cardiac activity and behavior, during two sessions of three consecutive routine handling sets. Measures of cardiac activity included mean heart rate (HR_mean_), and two measures of heart rate variability (HRV): the root mean square of successive differences between normal heartbeats (RMSSD); and the high frequency absolute power component of HRV, log transformed (HF_log_). We also assessed human handlers' emotional state during handling sessions following an intervention designed to reduce stress, compared with sessions conducted on a different day and following a control activity. Polar H10 cardiac sensors were used to simultaneously record cardiac activity for both canine and human participants, and behavioral data were collected via digital video. The strongest influence on the dogs' stress levels in our study was found to be increasing familiarity with the setting and the handler; HR_mean_ and SI decreased, and HRV (as RMSSD) increased, significantly from the first to the third handling set. Canine HRV (as HF_log_) was also highest in set 3, although the difference was not statistically significant. There were no strong patterns found in the human cardiac data across handling set, session, or by pre-handling activity. We did not find consistent support for emotional contagion between the dogs and their handlers in this study, perhaps due to the brief time that the dogs spent with the handlers. Recommendations for application to dog handling, and limitations of our methods, are described.

## Introduction

Despite their dedication to animal wellbeing, many animal-care and veterinary professionals suffer from high levels of work-related stress, and this can result in compassion fatigue, burn-out, clinical depression and other signs of chronic stress (1–6). Unresolved chronic stress in animal care workers can jeopardize both staff and animals, as staff may be insensitive to animal stress and inured to risks while handling highly-stressed animals, which may compromise the quantity and quality of the care these animals receive (7). Negative stress due to human handling has been documented in numerous domestic and laboratory animals including dogs, *Canis lupus familiaris* (8–15). Although regular veterinary visits are an essential part of caring for companion dogs, many dogs display significant stress at the veterinary clinic. In one recent survey-based study of >26,500 dog owners, over 50% of dogs were reported to display fear at the veterinary clinic, ranging from mild to intense (16). The environment and human interactions, rather than dog characteristics like breed or age, are likely to be the primary drivers of the prevalence and severity of this issue (16). On the other hand, positive interactions between friendly humans and shelter dogs have been shown to have a beneficial effect on dogs by reducing their stress level (17–20).

When assessing canine stress during human-animal interactions, it is important to note that domestic dogs can detect human emotion through visual, auditory, and chemical channels (21–23). Dogs have been reported to use social referencing with their human companions, with the emotional reaction of the human influencing that of the dog (24); and emotional contagion between humans and dogs has been reported, especially in female dogs and with duration of the relationship playing a role (25). Research suggests that factors such as owner personality, human-animal interactions, and choice of training methods, particularly over extended time periods, can all impact companion animal behavior and welfare (26–34). If, during human-animal interactions of shorter duration (such as in a veterinary clinic or animal shelter), the emotional state of the human can influence the emotional state of the animal, then emotional state of human caretakers may play an indirect role in animal welfare, in addition to any direct (behavior-based) impacts that may occur. The goal of the present study was to assess whether the emotional state of a human handler is associated with the emotional state of a dog during routine handling exercises, when the handler is unfamiliar to the dog, and the duration of the interactions brief. To help assess whether emotional contagion occurs in these circumstances, handling exercises were conducted before and after the addition of a mild verbal (psychological) stressor to the human. If human stress can be detected by the animals during direct interactions such as these, and thus impact the stress levels of these animals under veterinary or shelter care, any steps that could be taken to reduce or mitigate stressors for both humans and non-humans would seem advisable, in order to maintain high standards of care for companion animals (7, 11).

Stress levels of dogs and humans can be assessed in various ways, including behavioral measures (body language, behaviors indicative of stress) and physiological stress responses, such as measurement of stress hormones or changes in cardiac activity. Cardiac activity, particularly heart rate variability (HRV), has frequently been used as an indicator of stress and emotional state in human and non-human animals (9, 35–39).

HRV describes variations of instantaneous heartbeat intervals (i.e., the time intervals between successive heartbeats, aka RR intervals) and reflects changes in activity of the autonomic nervous system (ANS), which (among other functions) is the primary mechanism in control of the “fight-or-flight” response. HRV provides a reliable index of cardiac vagal tone, which represents the contribution of the parasympathetic branch of the ANS (responsible for the “fight-or-flight” response) to cardiac regulation (40) and is, thus, linked to emotion. Research supports that HRV may be a significant indicator of important body functions associated with stress, adaptability and health (41). In general, lower HRV at rest is correlated with stress, anxiety, worry or panic (42). HRV has also been widely used to assess stress and emotional state in animals in response to environmental variables (35). For example, Kuhne et al. (9) used real-time measurement of HRV to document increased emotional stress in dogs (indicated by increased heart rate, HR, and reduced HRV) exposed to certain types of handling. Changes in HRV can occur in the absence of detectable alteration in heart rate (35), and thus are considered a more reliable indicator of emotional state than heart rate alone. For example, in a study of lamb responses to aversive events, learning to control an aversive event was associated with elevated HRV, but was not reflected in heart rate; while lack of control over the environment was associated with a decreased HRV, suggesting greater sympathetic control over cardiac activity (36, 37). Therefore, methods for HRV analysis based on measurement of interbeat intervals, such as those used in this study, allow for a more detailed interpretation of cardiac activity in terms of ANS activity (43). Heart rate is continuously monitored and recorded during study interventions, using non-invasive electrodes placed on the animal's skin which continuously transmit data to the heart rate monitor; HRV parameters are then calculated from the recorded HR data, using specifically-designed software, for comparison between participant states (e.g., baseline vs. intervention).

HRV has been successfully used in the study of emotion in domestic dogs in a number of recent studies [e.g., (8, 9, 44, 45)]. Maros et al. (39) used a harness-mounted telemetric system for ambulatory measurement of RR intervals [ISAX (46)] on dogs, and reported that HR increased during activity; no changes were seen in HRV based on body position or movement. In that study, HRV did increase when dogs oriented toward a favorite toy (and when petting by an unfamiliar individual ceased), leading the authors to conclude that HRV could be a good indicator of the dogs' attentive state (39).

This study investigated relationships between emotional states and stress levels of human handlers and domestic dogs, using simultaneous measures of cardiac activity and behavior of human and dog during routine handling in a veterinary clinic setting, in both non-stressful and stressful handling contexts. In addition, the study assessed the impact of an intervention activity designed to reduce stress on the human handler's emotional state during handling. Cardiac activity and behavior during handling following the intervention were compared to these variables following a control activity not specifically designed to be calming. Our hypotheses were that (1) increased stress in the human handler would be reflected in increased stress in the dog being handled; and (2) an intervention designed to reduce the human handler's stress level would be reflected in lower stress levels in both the human and the dog during the subsequent handling session.

## Materials and Methods

All research protocols for this study were reviewed and approved by the University of California at Davis' Institutional Animal Care and Use Committee (IACUC), protocol approval #20756, and Institutional Research Board (IRB) for human subjects, protocol approval #1313227-1. For this study, 40 adult dogs and their owners were recruited from the university community. Healthy adult (>1 yr old) dogs of any breed and larger than 20 lbs (9.1 kg) were eligible to participate. The minimum size limit was put in place because it was difficult to fit the HR monitor chest strap to dogs <20 lbs in such a way as to allow consistent HR data from these dogs. Dogs were screened prior to inclusion in the study to ensure the safety of our research participants and student research assistants; dogs with a known history of excessive fear or aggression toward humans or other dogs were not included in the study. To maximize generalizability to companion dogs, dogs who have undergone unusually high levels of socialization and obedience training (such as working, assistance, or therapy dogs) were not included in the study. Participants were offered an incentive ($50 Amazon.com gift card) for completing the two data collection sessions.

**Figure 1:**
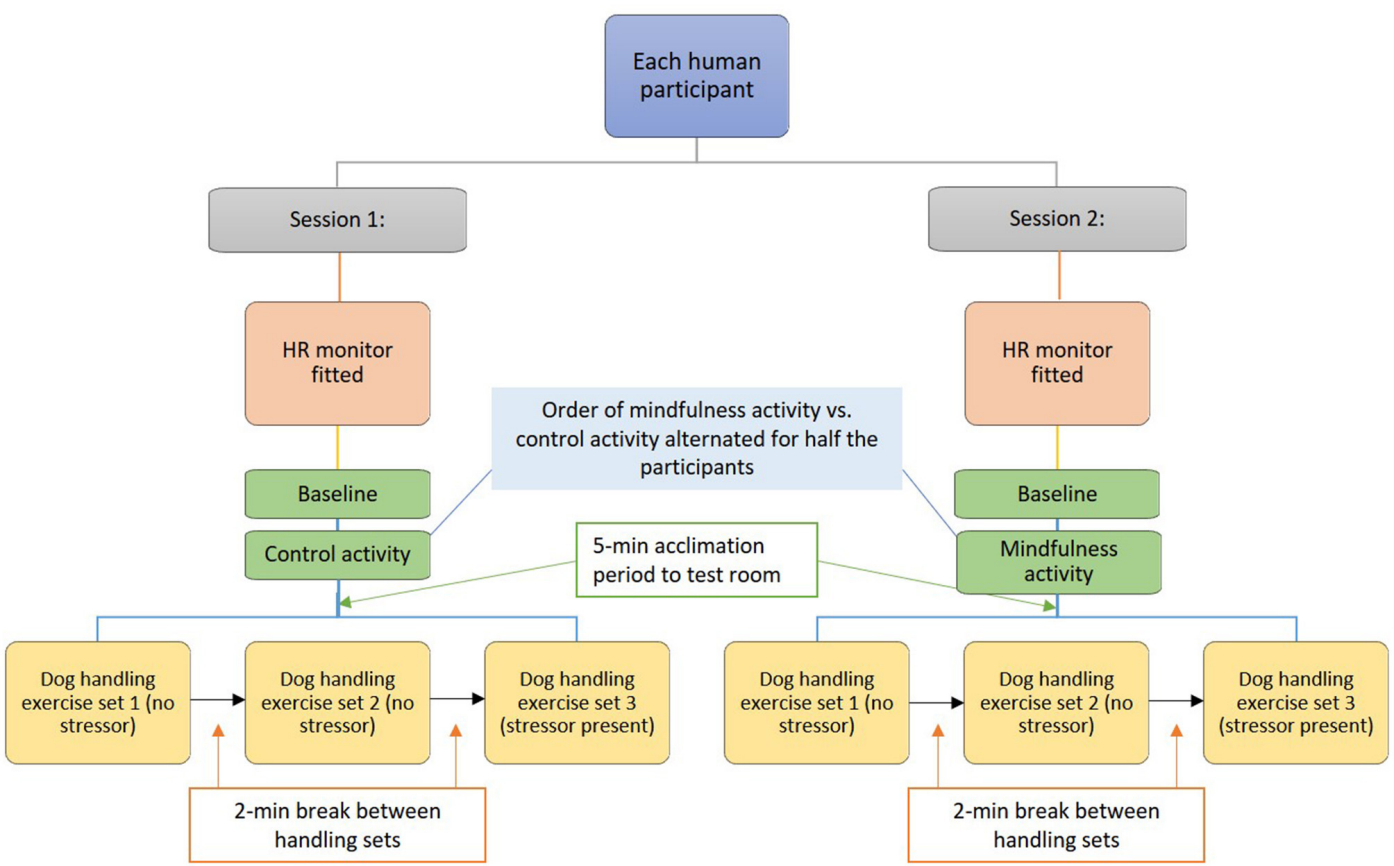
Sequence of data collection sessions and handling sets.

To investigate potential impacts of the handler's emotional state on the dog's emotional state, during each research session, each handler and dog pair completed three identical sets of handling exercises, with a short (2-min) break between sets (detailed description of these exercises is provided below). Just prior to the start of the third set of handling exercises, a mild verbal stressor was introduced by the researcher (Figure 1); the intent was to influence the emotional state of the handler, by putting the handler on alert for a potentially stressful or aversive event, without the stressor directly impacting the dog [see (47) for a similar experimental design involving horses].

To investigate potential impacts of an intervention designed to reduce animal handler stress on the outcomes of our experiment, each dog/human pair came in for two data collection sessions 1-week apart and at approximately the same time of day (Figure 1). Prior to direct interaction with the unfamiliar dogs, human participants participated in either a brief mindfulness meditation activity, or in a control activity (detailed description of the pre-handling activities is provided below). Mindfulness meditation activities have been shown to improve resilience in workers employed in high stress professions (48), to help with emotion and behavioral regulation (49), and to increase positive emotions (50). Order of participation in the control vs. mindfulness activities was alternated for a balanced cross-over design, such that half the study participants did the mindfulness activity in session 1, followed by the control activity in session 2 (1 week later); the other participants did these activities in reverse order.

### HR Monitor Fitting

Thirty minutes prior to the start of the handling exercise, dogs and humans were fitted with Polar H10 (Polar Electro Oy; Kempele, Finland) non-invasive cardiac sensors on Polar Pro soft straps (containing two electrodes). Polar HR monitors have been validated for use in reliably measuring HRV in stationary dogs (51), although some researchers have expressed concern about their validity and reliability compared to electrocardiogram (ECG) data, particularly when the animal is moving [for pigs (52); for horses (53)]. However, Essner et al. (54) assessed the use of Polar HR monitors on standing dogs and when dogs were in motion (trotting on a treadmill), and concluded that the criterion validity and instrument reliability of the Polar monitors were excellent, and the standard error of measurement was low (54). They noted that the measurement error was comparable to ECG, with the Polars both under- and overestimating HR, which may have particular importance in the clinical setting. Our goal in the present study, using a within-subject research design, was to use the Polar HR monitors to compare cardiac activity parameters of dogs (and humans) during handling. For our purposes, clinical accuracy of the Polar monitors in measuring cardiac activity was less important than instrument reliability, which is reported to be high (54), across our experimental conditions.

For the dogs, a strip of hair approximately 2 inches (5.1 cm) wide x 12 inches (30.5 cm) long was shaved from each dog's chest between the axillae (armpits), and ultrasound gel (Spectra 360; Parker Labs, Fairfield, NJ) applied between sensors and skin to ensure complete contact with the canine subject's skin, and allow continuous HR readings. The HR monitor and strap were then further secured using elastic veterinary bandage material (Vet Wrap; WildCow, Marietta, GA), to minimize electrode shifting during the dog's movement. Low-stress handling techniques were used for HR monitor fitting, to avoid unnecessary stress to the dogs. If any dog showed signs of marked distress and/or defensive aggression during the HR monitor fitting (or later, during handling exercises), the session was immediately ended and the dog returned to his/her owner. For humans, Polar H10 monitors on chest straps were fitted to human volunteers by the researchers, with ultrasound gel applied between the sensors and skin. The HR monitors were connected via Bluetooth® to Apple iPhone (Apple, Inc.; Cupertino, CA) smartphones running the Heart Rate Variability Logger app (Altini, M.; A.S.M.A. B.V.); after the data collection was complete, cardiac data files were uploaded via the file hosting service Dropbox™ (Dropbox, Inc.; San Francisco, CA) for storage and later analysis.

### Baseline Data Collection

After the HR monitors were securely fitted and connectivity to the smartphones confirmed, both canine and human participants were allowed 10 min to acclimate to wearing the HR sensor equipment; baseline cardiac activity data was collected during this time window. As human and canine participants could be both sitting and standing during the handling sets, the 10-min HR baseline time was divided into two segments; 5 min of participants sitting down, and 5 min standing. Canine participants were separated from their owners and spent 10 min with a trained research assistant, in a quiet area away from the testing room, during baseline data collection.

### Pre-handling Activities

Prior to beginning the handling exercises, human participants (without their dog present) participated in either a brief (10-min) mindfulness meditation activity, or in a 10-min control activity (either reading a short informational pamphlet on dogs, or engaged in a casual conversation on neutral topics with a trained student research assistant). The reading and conversation activities were briefly introduced by a research assistant, to better match the mindfulness activity setting, and were conducted in the same room as the mindfulness activity. For the mindfulness activity, following a 5-min introduction by a trained instructor, dog handlers were led in a 5-min guided mindfulness meditation that included breath focused attention; open monitoring of the transitory of sensory experiences, thoughts and emotions; and concluding with a mindful loving kindness meditation. All potentially emotion-inducing items (such as pet memorial brochures, artwork, etc.) were removed from the pre-handling activity room prior to use.

### Behavioral and HR Data Collection

Polar H10 HR monitors were used to continuously track HR (allowing calculation of heart rate variability, HRV, using associated software) of both handler and dog, during a session consisting of three short, nearly identical routine handling exercises (see below for differences).

To avoid the confounding effects of the previous relationship between owner/dog on the results (and, to increase applicability to interactions occurring in animal care settings such as shelters and veterinary clinics), two sessions, involving two handler/dog dyads, were conducted simultaneously, in separate rooms. Prior to the handling exercises, dogs were exchanged between participating owners, so that human handlers were not working with their own dog. Rooms used for this study were standard veterinary clinic exam rooms, located at the Center for Companion Animal Health (CCAH; School of Veterinary Medicine, University of California, Davis). All data collection sessions were conducted on weekends when the CCAH was closed, to minimize impacts of other dogs or human activity on the research participants. Data collection took place from April–December 2019, prior to Covid-19 pandemic restrictions and mask mandates.

Once the dog and handler were in the research study room, they were allowed 5 min to acclimate to the room (Figure 1). During the acclimation period, handlers were allowed soft verbal and physical contact with the dogs. After this acclimation period, participants were led (via verbal instructions) through the handling exercises by a trained research assistant. A second research assistant filmed all handling exercises using a Canon Vixia HF R800 HD digital camcorder (Canon, Inc.; Tokyo, Japan) for later analysis of behavioral data. Interactions were synchronized to the HR data by marking the exact (simultaneous) start time for both recordings, and/or by using the “event annotation” function in the HR data logger app. Research assistants present in the testing room could interact verbally with the human participants for the purposes of providing handling protocols, but were instructed to not interact directly with the dogs during data collection.

### Handling Exercises

All handling exercises were conducted on the floor, with the handler seated on a stool or on the floor. Dogs wore a flat buckle collar and a short leash; use of the leash was minimized during handling exercises. Handlers were instructed to use only low-stress handling approaches with the dogs (e.g., no use of force or firm restraint, no punishment of any kind, maintain gentle voice and handling; food treats were available to distract or lure dogs as needed). Handling exercises consisted of items 1 (“Look”), 2 (“Touch Sensitivity”) and 4 (“Squeeze”) from the ASPCA's SAFER behavioral assessment (55) (Table 1); each assessment item takes between 30 s and 2 min. If the handler completed the handling exercises within a given set in <2 min., they were asked to repeat the same exercises until the 2-min set time was reached. Three handling sets were performed during each research session. Following each handling set, there was a 2-min break period for the handler and the dog.

**Table 1:** Handling exercises performed during simultaneous cardiac and behavioral data collection.

**Handling exercise:**	**Description:**	Goal of exercise (from ASPCA^*^):
“Look”	• Place the chair in the room where it is not against a wall or in a corner. • Sit upright in the chair with knees apart approximately shoulder width and feet flat on the floor. • Quietly coax the dog into a position between the knees, facing the handler. • Gather up the leash and lightly grasp the collar with one hand, then lightly cup dog's lower jaw in both hands and encourage the dog with soft eye contact. • Allow the dog to pull away and/or avoid the eye contact. • If the dog moves his/her head before he/she has settled in your hands, repeat up to three times.	To determine how the dog responds when lightly restrained and given soft yet direct eye contact from a stranger.
“Touch sensitivity”	• Sit upright in the chair with legs moderately spread and feet flat on floor. • Coax the dog to stand perpendicular to handler, centered in front of handler's knees. The dog can stand or sit for this item. • If the dog's body is oriented to the right, gather the leash in your right hand and grasp the collar with your right hand, fingers facing toward the dog's rear. Brace your right elbow against your knee in order to control the dog's head. If the dog's body is oriented to the left, gather the leash in your left hand and grasp the collar with your left hand, fingers facing toward the dog's rear. Brace your left elbow against your knee in order to control the dog's head. • With your free hand, grasp (with pressure slightly more than normal touch) and lift and twist handfuls of skin and fur in a kneading motion, starting at the neck, following an inch or two outside the spinal column, working down the dog's body past the tail to the flank and back up again. • Execute the “down and back” pattern twice.	To determine the dog's touch sensitivity. Fearfulness toward new experiences may be noted as well.
“Squeeze”	• While standing in front of chair, coax dog so that he/she stands or sits perpendicular to the handler. • Sit with knees spread apart and feet on floor. • If the dog's body is oriented to your right, with your left hand gather excess leash and hold the collar, fingers facing upwards toward the dog's head. If the dog is oriented to your left, with your right hand gather excess leash and hold the collar, fingers facing upwards toward the dog's head. Brace the arm holding the leash and collar on your knee, pushing dog out from handler. • With the other hand, pick up foreleg nearest you midway down leg and lightly run hand down to paw. • Using just finger pads, squeeze between the dog's toe pads. Increase pressure on the skin between the 2 toes until the dog responds. Allow dog to withdraw paw. • If there is no response in 3 s, stop the pressure and release the paw. • Repeat for a second time, using the same paw.	To determine the dog's sensitivity response, bite inhibition, acceptance of being held or touched in a mildly controlled and unpleasant manner.

The three exercises were conducted in order, and comprised one handling “set;” three “sets” were conducted during each data collection session, with a 2-min break between sets. Exercise descriptions are adapted from the ASPCA's SAFER^*^ behavioral assessment protocols.

* *ASPCA (55)*.

Just prior to the start of the last of the three handling sets, the verbal stressor was introduced; two forms of added psychological stressor were used in this study. For the first 3 months of data collection, the handler was told that a research assistant might bring another unfamiliar dog into the testing room, and, as the dog they were handling was mildly dog reactive, they should be prepared for a negative reaction from the handled dog. This stressor was designed to put the handler on alert for a strong reaction from the dog they were handling, including the possibility of dog-dog aggression in the confined space of the testing room. No additional dog was brought into the room. In order to maintain the handler's belief that a dog might enter the room, the handlers were told that the days on which the additional dog would be brought into the room were randomly assigned (so, they might need to deal with the second dog on one, both, or neither of their two sessions). At the study midpoint, and based on preliminary analysis of behavioral data collected to date (comparison of canine stress levels in handling sets 1 and 2 vs. 3; see “Data analysis,” below), it was decided that this stressor might not be sufficient to significantly increase the stress level of the human participants. This was supported by anecdotal observations by the research team of human participants' lack of marked behavior change following introduction of the initial stressor. For this reason, for the remainder of data collection, an alternate anticipatory stressor was used: the handlers were told (again, just prior to beginning the third handling set) that their performance on the handling exercises in the third and final set would be evaluated by the research team using the video recordings, with the participant who performed the exercises the most accurately (and with no prompting from research assistants) awarded an additional $50 gift card. In both cases, the third handling exercise (post-stressor) was in fact identical to the previous two (pre-stressor), other than the introduction of a verbal stressor designed to influence the attitude/emotional state of the handler via increased psychological stress. It should be noted that the goal of the verbal stressors was not to mimic stressors typically found in the veterinary or shelter setting (although dog-dog aggression could be reasonably anticipated in either setting). The goal was simply to change the emotional state of the human handler, allowing us to compare the emotional state of the dogs under varying emotional states of the handler.

### Data Analysis (Cardiac Activity)

All cardiac (RR interval) data recorded via the Polar H10 sensors were pre-processed for artifact correction and trend removal using Kubios HRV, a device-independent software used for HRV analysis (62). The cardiac activity measures used in this study were HR (mean heart rate for handling set); one time-domain measure of HRV (RMSSD, the root mean square of successive RR interval differences, in ms); and one frequency-domain measure of HRV (HF, absolute power of the high frequency band). HF power was log transformed to increase normality of those data. RMSSD and HF were chosen for analysis as these are recommended for studies of psychophysiological research, as they may best reflect vagal tone (40). For all HRV variables, we used a within-subject approach by calculating the difference from baseline measurement (for each participant and session date). Within-subject designs have been highly recommended for psychophysiological and biobehavioral research, given the complex interactions influencing HRV and high inter-individual variation (40, 63). Difference from baseline was calculated as experimental value minus baseline value (so, if the experimental value was lower than the baseline value for that parameter, difference from baseline would be a negative value; if experimental > baseline, difference would be positive). As the primary position of both human and dog during the handling sets proved to be seated, the 5-min “sitting” baseline measurement was used for comparisons, when available. For some baseline measurements, it was not possible to divide the time equally into “sitting” vs. “standing” baselines (for example, due to a dog's unwillingness to remain seated calmly for 5 full min); in these cases, the mean baseline measurement (for the 10-min baseline) was used. Note that no significant differences were found in the baseline “sitting” and overall mean baseline HR measurements, for either humans or dogs, in either session 1 or 2 (all *p* > 0.355).

### Data Analysis (Behavioral)

Behavioral data were coded from the digital video recordings using event-logging software [BORIS (56)]. Frequencies and durations of established canine stress-related behaviors [e.g., lip-licking, tucked tail, panting, “whale eye” (57–60)] were tabulated (Table 2A). Times when the dog and/or human were out of view of the camera were excluded from the analysis. Despite attempts to keep set duration consistent, the time it took for a handler to complete each handling exercise varied, as did the amount of time the dog and/or human were “in view” of the digital video recording device for a given handling set. Thus, the behavioral data was converted to rate per minute, to allow comparison between sets and across data collection sessions. For each set, duration (in seconds per minute) was calculated for all state behaviors (e.g., sitting, lying down); frequency (in number of occurrences per minute) was calculated for all event behaviors (e.g., lip lick, yawning). A canine “stress index” (SI; rate of stress behaviors observed/min) was calculated for each handling set as the sum of both state and event behavior rates for that set. Frequencies and durations of human behaviors indicative of stress [e.g., facial expressions such as frowning, furrowed brow; self-directed behaviors such as covering face or mouth; (61)] were also recorded (Table 2B).

**Table 2:** Ethograms used for tabulating participant behaviors from the digital video recordings canine **(A)** and human **(B)**.

**(A). Canine ethogram**.		
**Behavior code**	**Behavior type**	**Description**
Lying down	State	Dog is lying down on side
Standing up	State	Dog standing still, all four paws on floor
Moving	State	Dog is changing locations while moving (not just moving in place)
Rolling^*^	State	Dog rolls onto back
Sitting	State	Dog sitting down
Out of view	State	Any part of the dog is not visible on screen
Wag	State	Dog wags tail
Tail tucked^*^	State	Dog's tail is tucked between legs
Gaze at handler	State	Directional look by dog toward handler
Whale eye^*^	Event	Dog's eyes widen; white parts of eye exposed
Yawn^*^	Event	Dog Yawns
Attention seeking	Event	Dog voluntarily initiates physical contact with participant. Code begins when physical contact is first made.
Snap or bite^*^	Event	Dog snaps or bites at handler
Jump	Event	Dog jumps up on handler or researcher
Shake^*^	Event	Dog shakes head or body
Lip lick^*^	Event	Dog licks lips
Panting^*^	State	Dog is visibly panting
Vocalization^*^	Event	Dog vocalizes
**(B). Human ethogram**.		
Standing up	State	Standing Up
Moving	State	Changing location in room through movement
Sitting	State	Sitting down on stool, sitting, or kneeling on ground
Out of view	State	Any part of the human is out of frame
^*^Negative facial expression *Modifiers*: Frown Grimace Bite/Chew/Licks Lips Eyes Widen Rapid glances around room Rapid blinking	Event	Participant makes negative facial expression or eye movement. Note: a frown consists of brows coming together and lips are pulling downward
Positive facial expression *Modifiers*: Smile Laugh	Event	Participant makes positive facial expression
^*^Self-directed behavior *Modifiers*: Touching head/face Crosses arms across body, self, hug Other self-directed activity	Event	Human touches self, covers mouth or face with hands, etc.

Stress behaviors used in calculating the stress index (SI) are indicated with an asterisk^*^. Note that not all behaviors in the ethogram were observed during the study.

### Statistical Analyses

Initial estimates for sample size for this study were made according to recommendations of Ruxton and Colegrave (64), to meet or exceed sample sizes in published studies successfully able to answer similar research questions, in this case involving HRV analyses in dogs and other mammals [e.g., *n* = 7 (38); *n* = 14 (39); *n* = 20 (18); *n* = 24 (9, 43); *n* = 27 (51); *n* = 30 (36)]. In addition, we ran an a priori power analysis (G^*^power v.3.1.9.2) for a repeated measures ANOVA (input parameters: effect size = 0.20, α = 0.05, power = 0.80); calculated required sample size was *n* = 36, supporting our proposed sample size of 40 dogs.

For both human and canine participants, to investigate potential impacts of the introduction of the verbal stressor and/or the pre-handling activity on emotional state, repeated measures ANOVAs were used to look for differences in the cardiac parameters by handling set (1, 2, or 3), with session (1 or 2) and pre-handling activity (mindfulness vs. control) as fixed effects. Although human participants' HR (mean) values were slightly higher, and their HRV variables slightly lower, during handling set 3 following the introduction of the alternate verbal stressor, the differences between the cardiac variables when the original vs. alternate stressor were used were not statistically significant (HR: *p* = 0.216; RMSSD: *p* = 0.968; HF: *p* = 0.608). For this reason, no further distinction was made in the statistical analyses between data collected using the first, vs. second, verbal stressor. *Post-hoc* pairwise comparisons between groups using Tukey's HSD were conducted to better understand within-subject and between-subject patterns in the data. Standardized effect sizes for the repeated measures ANOVAs were estimated using partial eta-squared ($\eta_{\text{p}}^{2}$), with 0.01 = small, 0.06 = moderate, and 0.14 = large effect size. Simple effect sizes are demonstrated with mean differences (±standard deviations) between groups. In addition, Pearson's correlations for paired samples were calculated to investigate relationships among and between the human and canine cardiac parameters.

As the behavioral data were non-normal (based on Shapiro-Wilks tests for normality), Friedman tests (a non-parametric alternative to repeated measures ANOVA) and Mann-Whitney U tests were used to compare SI by handling set, by session, and by pre-handling activity. For the non-parametric analyses, *post-hoc* pairwise comparisons were conducted for the Friedman test using the Nemenyi test (65), and for the Mann-Whitney U tests and correlation matrices using Bonferroni corrections for multiple comparisons. Standardized effect sizes for the Friedman test were estimated using the Kendall's W value (66); the Kendall's W coefficient uses the Cohen's interpretation guidelines (0.1 - <0.3 = small; 0.3- <0.5 = moderate; ≥0.5 = large). Pearson's correlations for paired samples were also run to assess relationships between the behavioral (SI) and cardiac parameters. All statistical analyses were conducted in XLSTAT 2021 (Addinsoft, Inc, New York, NY, USA) for Microsoft Excel (Microsoft Corp., Redmond, WA, USA), with α = 0.05.

## Results

**Figure 2:**
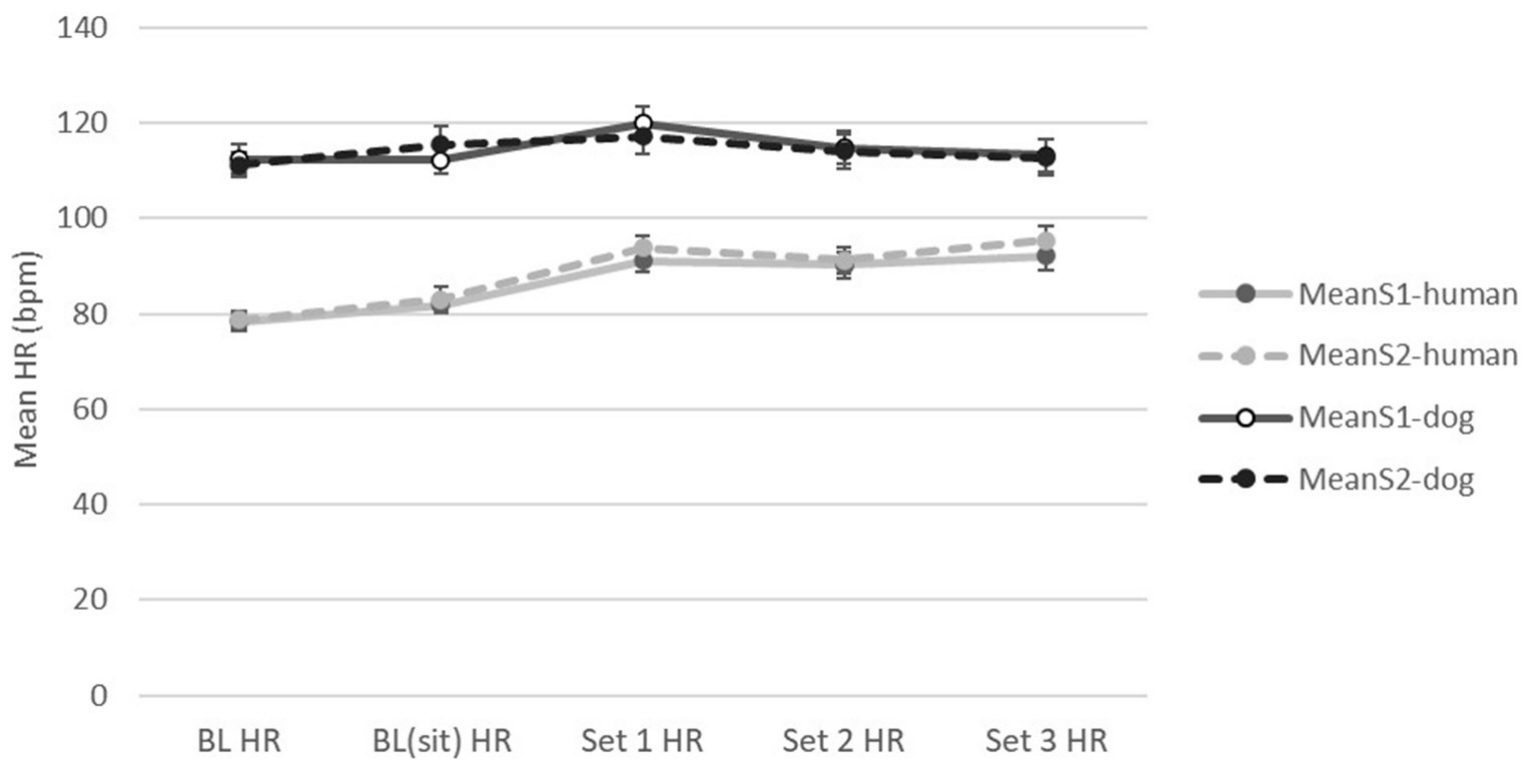
Mean HR for canine and human participants for data collection sessions 1 and 2, during baseline and the three handling sets. As the majority of the handling exercises were conducted while the human and dog were seated, the baseline data when sitting [BL(sit)] were used for calculation of “difference from baseline” when available. When BL(sit) data were not available, baseline data for the entire baseline period (BL), including both sitting and standing, were used. No significant differences were found in the baseline “sitting” and overall mean baseline HR measurements, for either humans or dogs, in either session 1 or 2 (all *p* > 0.355).

Forty healthy adult dogs were recruited for this study; two of the dogs were released from the study due to excessive fearfulness when handled, leaving 38 dogs and their human caretakers participating in the handling sessions. Breed designations were provided by the dogs' owners; half of the dogs (*n* = 19, 50%) were mixed breed; 11 breeds were represented, including Golden Retriever, Labrador Retriever, Boxer, Beagle, and others. Dogs' ages ranged from 1 to >10 years (mode age category: 1–2 yrs), and weight ranged from 20.1 to >80 lbs (mode category: 40.1–50 lbs, or 18.2–22.7 kg). Sixteen dogs (42.1%) were female/female spayed; the remainder were male/male neutered. All human participants in this study were students or graduate students at the University of California, Davis, ranging in age between 18 and 44 years (mode category: 18–24 yrs). The overwhelming majority of human participants were female (*n* = 36, 94.7%). Three human participants only completed session 1 of the study protocols; data for these participants were excluded from analyses where appropriate. A number of cardiac activity data files were lost due to equipment issues: temporary losses of Bluetooth® connectivity between the sensors and the smartphones during data collection, and technical issues with uploading for storage and later analysis. Final participant counts used in the cardiac activity data analyses were 30 humans (session 1), 26 humans (session 2), 31 dogs (session 1), and 26 dogs (session 2). Mean HR for canine and human participants during baseline and the handling sets, and for data collection sessions 1 and 2, are shown in Figure 2.

**Figure 3:**
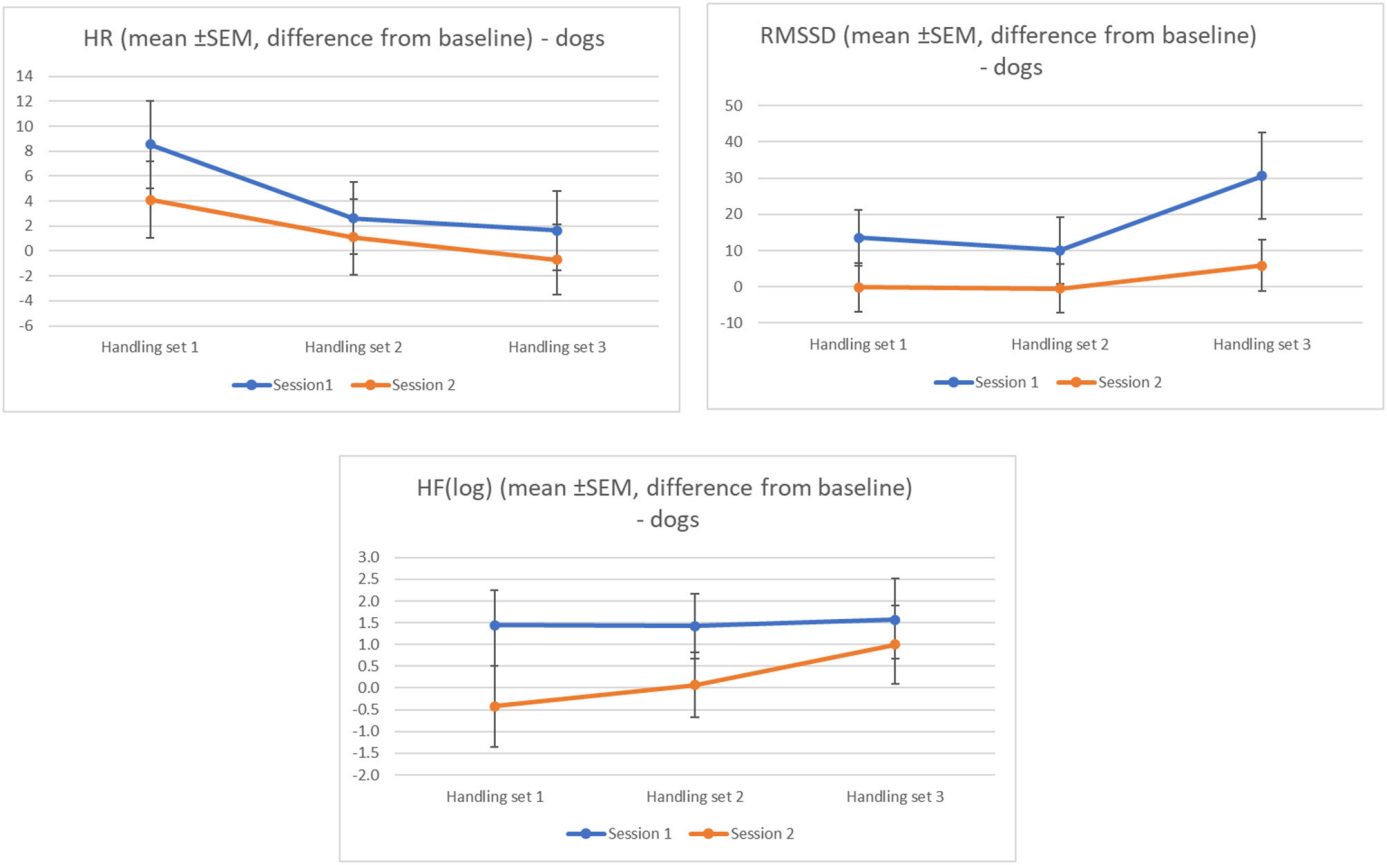
Canine cardiac activity variables by session number (1 vs. 2) and handling set number (1-3). Statistical significance for individual comparisons (by session, and by handling set) can be found in the text, and in the Supplementary Material.

**Figure 4:**
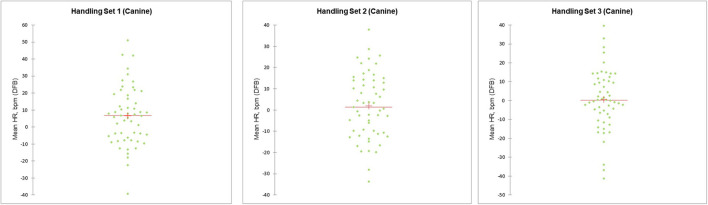
Scatterplot illustration demonstrating individual variation in canine cardiac responses (HR, difference from baseline) to handling, during the three handling sets. The horizontal line on the graphs represents the mean, and can be compared to the “0” point on the y-axis (representing no difference from baseline HR).

For the dogs, there was a significant difference in mean HR between the handling sets (*F*_(2,101)_ = 4.203, *p* = 0.017; $\eta_{\text{p}}^{2}$ = 0.08); mean HR values and differences (± SD) from baseline for each handling set are shown in Table 3A. There were no differences in mean HR by session, or by pre-handling activity, nor any interaction effects. The ANOVA table results are available in the Supplementary Material 1. Overall mean HR was slightly higher than baseline in set 1, progressively declining to near baseline by set 3 (Table 3A). In session 2, mean HR fell below baseline by handling set 3. Although not significant, mean HR was consistently higher during session 1 than session 2, for all three handling sets (Figure 3). Canine cardiac response varied by individual dog, with some dogs exhibiting reduced HR compared to baseline, others increased HR compared to baseline (Figure 4).

**Table 3:** Descriptive statistics for the canine cardiac activity parameters (raw data, and difference from baseline, BL), by handling set#: (**A)** mean HR, **(B)** RMSSD, **(C)** HF (log).

**Variable**	**Observations**	**Minimum**	**Maximum**	**Mean**	**Std. deviation**
**(A). Mean HR (canine)**.					
Set 1 Mean HR (diff from BL)	54	−39.307	51.057	6.484	17.393
Set 2 Mean HR (diff from BL)	54	−33.725	37.854	1.962	15.416
Set 3 Mean HR (diff from BL)	54	−41.215	39.583	0.601	15.844
Set 1 Mean HR	57	81.167	175.123	118.130	21.272
Set 2 Mean HR	57	80.363	163.277	114.102	19.760
Set 3 Mean HR	57	75.616	161.821	112.898	20.322
**(B). RMSSD (canine)**.					
Set 1 RMSSD (diff from BL)	51	−88.085	89.020	6.872	37.586
Set 2 RMSSD (diff from BL)	51	−73.048	225.547	3.641	41.993
Set 3 RMSSD (diff from BL)	51	−80.726	212.405	16.670	51.657
Set 1 RMSSD	57	9.318	163.360	60.812	37.572
Set 2 RMSSD	57	7.216	253.594	63.617	42.479
Set 3 RMSSD	57	6.418	250.639	75.058	53.440
**(C). HF (log) (canine)**.					
Set 1 HF (log) (diff from BL)	52	−8.711	8.596	0.537	4.393
Set 2 HF (log) (diff from BL)	52	−8.711	10.787	0.673	4.021
Set 3 HF (log) (diff from BL)	52	−8.150	10.208	1.169	4.813
Set 1 HF (log)	57	0.000	8.833	5.150	2.913
Set 2 HF (log)	57	0.000	10.787	5.180	3.106
Set 3 HF (log)	57	0.000	10.938	5.615	3.050

*A negative mean “diff from BL” value indicates an overall decrease in that value from baseline to experimental. Note that sample sizes for the raw data are larger where some baseline data, necessary for calculating “difference from baseline,” were lost due to technical issues*.

There was a significant difference in canine HRV (as RMSSD) between the handling sets (*F*_(2,95)_ = 3.219, *p* = 0.043; $\eta_{\text{p}}^{2}$ = 0.06), with RMSSD markedly higher in handling set 3; mean RMSSD values and differences (±SD) from baseline for each handling set are shown in Table 3B. RMSSD tended to be higher for session 1 than for session 2 for all three handling sets, but was not significantly different by session (*p* = 0.134) (Figure 3). There was no significant difference in canine RMSSD by pre-handling activity, nor were there any significant interaction effects found (Supplementary Material 1). As with HR, canine cardiac response varied by dog, with some dogs exhibiting reduced HRV (RMSSD) compared to baseline, others increased HRV (RMSSD) compared to baseline.

There were no significant differences in canine HRV (as HF) by set, session, or pre-handling activity. Mean HF(log) values and differences (±SD) from baseline for each handling set are shown in Table 3C. The interaction effect for canine HF between session^*^pre-handling activity^*^repetition approached significance (*p* = 0.065) (Supplementary Material 1). As with RMSSD, HF(log) tended to be slightly higher during session 1 than session 2, but not significantly (Figure 3). As with the other two measures of canine cardiac activity, response to handling varied by individual dog, with some dogs exhibiting decreased HF relative to baseline during handling, while others exhibited increased HF during handling.

**Figure 5:**
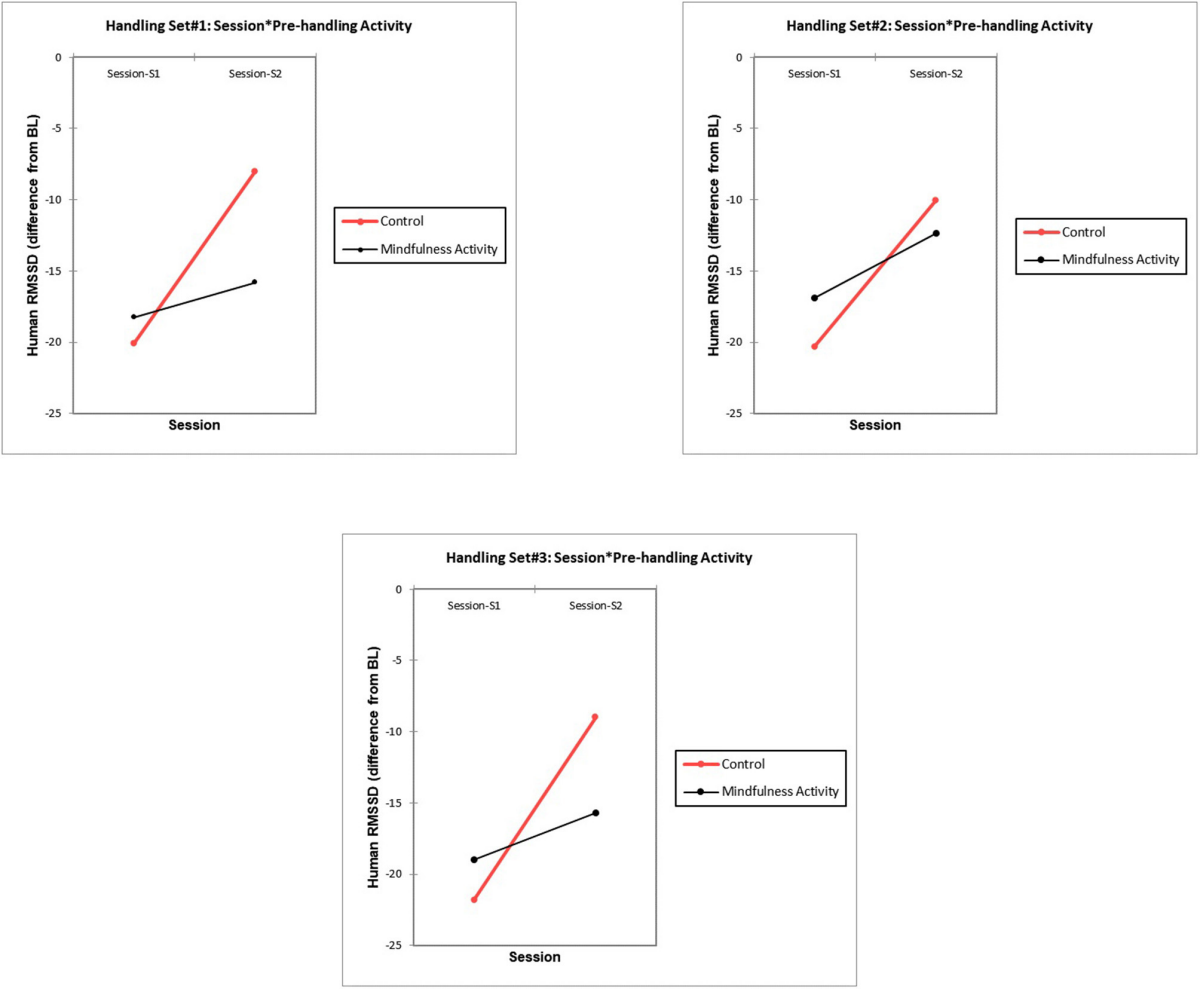
Means plots for RMSSD (human) for the three handling sets, by session (1 vs. 2) and pre-handling exercise (mindfulness vs. control), illustrating the significant interaction effect between session and pre-handling activity. Statistical significance for the interaction effect can be found in the text, and in the Supplementary Material.

For the human participants, HR (mean) tended to be higher relative to baseline during handling set 3 (post-stressor) than in sets 1 or 2 (pre-stressor) (Table 4A), although the difference did not reach statistical significance (*p* = 0.100). There were no significant differences in HR (mean) by session or pre-handling activity, and no significant interaction effects (Supplementary Material 2). Similarly, there were no significant differences in human HRV (as RMSSD) by set, session, or pre-handling activity; there was one significant interaction effect between session^*^pre-handling activity^*^set# (*F*_(2,95)_ = 3.202, *p* = 0.044; $\eta_{\text{p}}^{2}$ = 0.06) (Supplementary Material 2; Figure 5). Unlike the dogs, human results indicated consistently elevated HR and decreased HRV relative to baseline throughout the data collection (Tables 4A–C). Means plots of human RMSSD by set, session, and pre-handling activity (Figure 5) illustrate that when handling followed the control activity, human HRV (as RMSSD) was higher during session 2 than session 1; HRV following the mindfulness activity was more similar (and between the control high and low values), regardless of whether the mindfulness activity took place before session 1 or 2. There were no significant differences in human HRV (as HF) between set, session or pre-handling activity, and no significant interaction effects between any of the factors (Supplementary Material 2).

**Table 4:** Descriptive statistics for the human cardiac activity parameters (raw data, and difference from baseline, BL), by handling set#: **(A)** mean HR, **(B)** RMSSD, **(C)** HF (log).

**Variable**	**Observations**	**Minimum**	**Maximum**	**Mean**	**Std. deviation**
**(A). Mean HR (human)**.					
Set 1 Mean HR (diff from BL)	51	−14.744	39.104	11.226	11.076
Set 2 Mean HR (diff from BL)	51	−8.169	46.232	11.038	11.791
Set 3 Mean HR (diff from BL)	51	−11.183	82.405	14.337	16.611
Set 1 Mean HR	58	65.560	130.922	92.559	13.345
Set 2 Mean HR	58	60.879	140.689	91.743	15.031
Set 3 Mean HR	58	65.072	148.145	94.636	17.367
**(B). RMSSD (human)**.					
Set 1 RMSSD (diff from BL)	51	−157.252	55.967	−15.977	28.519
Set 2 RMSSD (diff from BL)	51	−161.652	42.008	−15.066	28.081
Set 3 RMSSD (diff from BL)	51	−163.598	35.856	−16.771	29.587
Set 1 RMSSD	58	4.700	111.945	30.752	19.159
Set 2 RMSSD	58	5.382	97.986	32.432	20.434
Set 3 RMSSD	58	4.105	83.529	30.291	18.085
**(C). HF (log) (human)**.					
Set 1 HF (log) (diff from BL)	51	−8.730	5.711	−1.834	2.802
Set 2 HF (log) (diff from BL)	51	−8.730	5.864	−1.686	2.906
Set 3 HF (log) (diff from BL)	51	−8.730	6.458	−1.513	3.371
Set 1 HF (log)	58	0.000	7.672	3.739	2.380
Set 2 HF (log)	58	0.000	7.645	4.014	2.516
Set 3 HF (log)	58	0.000	7.787	4.012	2.621

*A positive mean “diff from BL” value indicates an overall increase in that value from baseline to experimental. Note that sample sizes for the raw data are larger where some baseline data, necessary for calculating “difference from baseline,” were lost due to technical issues*.

Pearson's correlation analyses for paired samples revealed that the canine cardiac activity measures were significantly correlated, for both session 1 (HR and RMSSD: *r* = −0.265, *p* = 0.021; RMSSD and HF: r = 0.528, *p* = 0.0001), and session 2 (HR and RMSSD: r = −0.554, *p* = 0.0001; HR and HF: r = −0.446, *p* = 0.0001; RMSSD and HF: r = 0.548, *p* = 0.0001) (corrected α = 0.01). Human cardiac activity variables were also significantly correlated, as follows: for session 1 (HR and RMSSD: r = −0.303, *p* = 0.008; RMSSD and HF: r = 0.637, *p* = 0.0001), and for session 2 (HR and RMSSD: r = −0.376, *p* = 0.002). No significant correlations were found between the human and canine cardiac activity parameters during session 1; during session 2, canine RMSSD was significantly correlated with human HF (r = 0.432, *p* = 0.0001).

**Figure 6:**
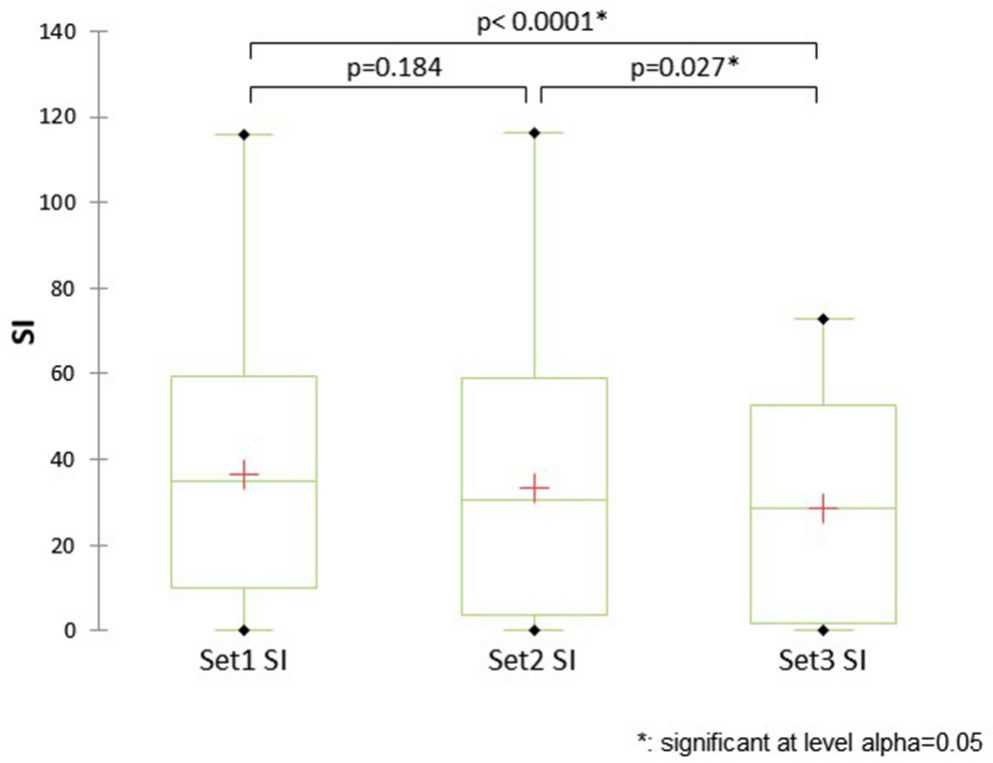
Comparison between canine stress index by handling set.

For the behavioral data, there was a significant difference between canine SI between handling sets (Q = 19.788, df = 2, *p* < 0.0001; Kendall's W = 0.14), with SI lower during set 3 than during sets 1 or 2 (Figure 6). As canine SI differed by handling set, comparisons between canine SI in sessions 1 and 2, and between the two pre-handling activities, were run separately for handling sets 1-3, using Mann-Whitney U tests. During handling sets 1 and 2, mean canine SI was slightly higher during session 1 than session 2, and in all three sets, slightly higher following the mindfulness activity; but the differences were not significant for any of the handling sets (all *p* > 0.20; corrected α = 0.008). There were very few human stress signs recorded in the behavioral data, so it was not possible to calculate a meaningful human stress index (SI) for behavioral analysis of the human participants.

Canine SI was not significantly correlated with any of the cardiac activity variables, in any of the handling sets. The correlation between SI and HF during handling sets 1 and 3 approached significance (set 1: *p* = 0.049; set 3: *p* = 0.026), but did not meet the corrected α = 0.017 for this analysis in either case.

## Discussion

In this study, canine cardiac activity parameters HR and RMSSD differed by handling set. Heart rate decreased as sets progressed from 1 to 3, and HRV (as RMSSD) was consistently higher in set 3 than during set 1, suggesting that dogs became less stressed as they became more familiar with the protocols and handler. HRV (as HF) also tended to increase across handling sets, although not significantly. Although the direction of change was similar between the two HRV measures, the reason for this difference in statistical significance between the HF and RMSSD results is not clear. Calculated standardized effect sizes for these comparisons were moderate. We found no evidence that the verbal stressor to the human handler impacted the dogs' stress levels in any way, nor that the handlers' participation in a mindfulness (vs. control) activity impacted the dogs during handling. Rather, our results suggest that familiarity with the handler and/or handling protocols was the factor most strongly influencing the dogs' emotional state during handling (bearing in mind that low-stress handling approaches were used throughout the handling exercises; e.g., no physical force, firm restraint, scolding or punishment were used). This is perhaps most clearly seen in the canine HR (mean) data across successive handling sets: HR was slightly elevated during the first handling set (relative to baseline) but dropped below baseline for handling set 2 and continued to decline for handling set 3. This conclusion is also supported by the behavioral data: dogs' stress levels (SI) were significantly lower during handling set 3 (post-stressor) than during sets 1 or 2 (pre-stressor), although the standardized effect size for the behavior results was small.

Heart rate tended to be higher during the first data collection session than the second, which lends some support to the importance of familiarity on stress reduction in these dogs, although this trend was not statistically significant. Both measures of HRV (RMSSD and HF) also tended to be higher during the first session, although as with HR, these differences were not statistically significant. The HRV results by session are somewhat surprising as, in contrast to the HR results by session, they would seem to suggest that the dogs may be less stressed (i.e., displaying higher HRV) during the first data collection session. However, Zupan et al. (45) studied beagle dogs' reactions to a series of different emotion-inducing stimuli (such as favorite vs. less-preferred food items, or social interactions with a familiar vs. unfamiliar human), and suggested that a combined decrease in RMSSD and HF may actually reflect an increase in the dogs' positive emotions, even when the dog is already in a positively-valenced emotional state. Although not statistically significant, a combined decrease in RMSSD and HF was seen in our study when comparing session 1 to session 2; this combined decrease was not seen for RMSSD and HF when comparing cardiac activity across handling sets within a session (see, for example, data presented in Figure 3). If Zupan et al. (45) conclusions apply here, this could explain the apparent contradiction between the HR and HRV results for the dogs in our study for the session data, and would support that familiarity with the handler and protocols was the strongest influence on the dogs' emotional state (particularly across handling sets within a given session, where the only statistically-significant differences were found).

Behavioral signs of canine stress (SI values) were also slightly lower during session 2 than 1 (although this latter difference was not statistically significant). Reduced stress when working with familiar individuals and with familiar low-stress procedures in the veterinary setting has also been reported for cats (67). For all cardiac activity parameters, there was considerable individual variation in the dogs' response to handling, with some dogs showing signs of increased stress relative to baseline (e.g., increased HR, decreased HRV), while other dogs showed decreased stress levels during handling (e.g., decreased HR, increased HRV). Individual variation in behavior is commonly seen in studies of dogs and many other species, and can play an important role in welfare (68, 69); we suspect (given the identical handling exercises, conducted in the same exam rooms, for all human/dog dyads) that each dog's prior and varying experiences with veterinary care may have been a strong driver of these differences, along with their varying comfort levels when interacting with unfamiliar humans (which in turn is shaped by temperament, previous experiences, etc.).

In addition, there were no strong patterns found in the human cardiac parameters across handling set, session, or pre-handling activity. Although HR (relative to baseline) of the handlers tended to be higher during set 3 (post addition of the verbal stressor) compared to sets 1 and 2, neither HR nor HRV differed significantly by handling set. This suggests that our attempts to increase human handler stress by introducing a verbal stressor prior to set 3 were not sufficiently impactful to be observable over any increased stress associated with participation in the novel study protocols (indicated by the increased HR, and decreased HRV, relative to baseline seen in the human cardiac results). A recent study assessing emotional contagion between dog and human (25) used an established method of causing social stress to the human participant [the Trier social stress test, TSST (70)], requiring participants to engage in a brief public speaking activity, followed by verbal mental arithmetic critiqued by an audience. In comparison to the TSST, our “stressor” was very mild; it may not have been particularly stressful for the human participants, or not stressful enough to cause any changes perceived by the dogs. We recommend that future studies of emotional contagion in a veterinary or shelter setting use an established experimental stressor to the human participants, perhaps increasing the length of time between repetitions, and using the last break between sets to apply the more salient stressor (in the absence of the dogs) to the humans.

A number of studies have reported physiological stress-reduction benefits of the presence of dogs [reviewed in (71)]. The degree to which any stress experienced by our volunteer participants in our study (all dog owners) was moderated by the presence of an unfamiliar pet dog is not known, although mean HR did not decline as the handling sets progressed for the humans as it did for the dogs. The human cardiac values did not differ significantly following the pre-handling mindfulness activity, although the interaction effect for HRV (RMSSD) between session^*^pre-handling activity suggests that the mindfulness activity may have had a mild stabilizing effect on handler emotional state, reflected in more consistent values of HRV regardless of whether the mindfulness activity preceded session 1 or session 2. As noted above, human participants did not overtly display many behavioral signs of stress, perhaps because of the presence of the two research assistants and the video camera in the testing room. Thus, we were unable to compare human SI across sets, sessions or pre-handling activity.

Although numerous within-species correlations were seen among the cardiac variables, we only found one significant correlation, during session 2, between the canine and human cardiac variables: canine HRV (RMSSD) was positively correlated with human HRV (HF), supporting that the more relaxed the handler, the more relaxed the dog, during the second session. However, given the lack of consistent correlations between the canine and human cardiac variables, combined with the differences between the canine and human patterns by handling set and session (e.g., human HR tended to be highest in handling set 3, post stressor; whereas canine HR was lowest during handling set 3), we could not demonstrate strong support for a relationship between human and canine stress levels in our study. The impact of social referencing between humans and dogs on dog behavior appears to be stronger when the human is familiar to the dog (24), for example when the human is the dog's owner or caretaker vs. a stranger, and perhaps influenced by the dog's learned ability to associate emotions of a familiar human with a given outcome (positive or negative) (72). Synchronization in long-term stress levels has been reported between companion dogs and their human guardians (73). Katayama et al. (25) found evidence of emotional contagion between humans and dogs based on analyses of human and canine HRV parameters (R-R intervals, SDNN and RMSSD), but reported that the strength of contagion was dependent on the length of the relationship (e.g., length of ownership) between the dog and the human. Lack of familiarity between handler and dog in our study may have played a role in the lack of consistent correlation between human and cardiac variables.

Reasons for the lack of correlations between the canine stress index (SI) and the cardiac activity parameters analyzed in this study are unclear. Canine behaviors chosen in this study to indicate stress in the dogs are widely recognized as canine stress signs. The most commonly-observed behaviors in this study were lip licking (constituting 76.7% of total frequency of event behaviors recorded), vocalization (18.0% of event behaviors), panting (82.8% of total duration for state behaviors), and tail tucked (16.6% of duration for state behaviors). Not surprisingly (given our study protocols), other signs of marked stress in the dogs such as attempting to snap or bite were rarely recorded (constituting < 1% of all event behaviors). Some canine behaviors can have multiple meanings depending on context. It is unlikely that the dogs in this study were panting due to heat exertion (given the study protocols, and the climate-controlled rooms used for data collection), and thus the most probable reason for panting was anxiety. Similarly, in this veterinary exam room context, lip licking was likely a reliable sign of stress in the dogs (74). On the other hand, vocalizations could have been a sign of distress in this context (e.g., due to separation from the familiar owner), but could also have been a sign of excitement (for example, due to receiving positive attention from a novel human). In addition, the ability to reliably code all the stress behaviors in the ethogram varied when using the digital video. For example, “whale eye” could only be coded when the dog's face was clearly visible in the video, and the affective state of “rolling on back” (submission vs. playful attention-seeking) was sometimes difficult to discern. Finally, as evident in the marked individual variation seen in the dogs' cardiac activity data, there was undoubtedly a great deal of variation in the dogs' perception of the stressfulness associated with the handling protocols, the study location (a veterinary clinic exam room), and direct physical interaction with an unfamiliar human. Veterinary visits are stressful for many dogs, and dogs' individual reactions to the veterinary setting and associated handling will vary based on a number of factors, as noted above, including (but not limited to) previous experience and environmental factors associated with a particular clinic (16, 75). These issues collectively may have made it difficult to discern clear and consistent associations between the behavioral and cardiac data.

This study was subject to a number of additional limitations. The first was the small sample size given the complexity of our study design and analyses. Although we were confident that our initial planned sample size of 40 human/dog pairs would be sufficient, two dogs were removed from the study due to excessive stress, three human participants only completed the first data collection session, and a number of cardiac activity data files (both human and canine) were lost due to the technical issues involving Bluetooth^®^ connectivity and data upload. The resulting sample sizes were lower than our goal: 26–30 humans, and 26–31 dogs, depending on session. In order to enter all three potential influences (handling set, session, and pre-handling exercise) into our models simultaneously, participant data from session 2 were considered independent from data from session 1; handling sets were designated as repetitions in the repeated-measures ANOVAs. Although there was a 1-week period and a difference in pre-handling activity between the two data collection sessions for any given participant (and corrections for multiple comparisons were used), this means that two sessions from the same participant were entered into the models as independent samples, and thus conclusions from the significant repeated measures ANOVAs on the cardiac parameters should be interpreted with caution. In addition, both humans and dogs moved frequently during the handling sets. Although Polar monitors have performed well on ambulatory animals in some studies [e.g., (58)], many authors have noted that movement can both influence HRV directly, and negatively impact the accuracy of HRV data collection (via production of artifacts). Thus, movement may “cloud the regulation linked to cognitive, emotional, social and health processes” [(40), p. 10]. We did not control for physical activity, such as acceleration or respiration, in this study. While some authors have recommended controlling for respiration to accurately assess vagal function [e.g., (76)], other authors recommend against correcting HRV for respiration in cases of participants' breathing spontaneously (as in our study) [summarized in (40)]. The design of our study meant that activity and respiration rates were likely to be similar across the experimental tasks (sessions and handling sets), given the identical handling exercises conducted throughout (and in the same limited space and layout provided by the two exam rooms used), and as suggested by Laborde et al. (40). Our within-subject study design may also have helped control for individual differences in subjects' innate tendency to move during the handling exercises. Finally, differences in duration of baseline periods used (5 min sitting vs. 10 min complete baseline) in the calculations of “difference from baseline” mean that the time-domain HRV measure (RMSSD) results should be interpreted with caution.

In conclusion, findings from this study support that even short-term familiarity with a previously-unfamiliar handler when low-stress handling protocols are employed (no use of force and only minimal restraint via leash, calm behavior by handler, availability of food treats for interested dogs, exercises conducted on the floor vs. the examination table) is associated with reduced stress in dogs in a veterinary setting. This suggests that, when dogs are in stressful settings such as animal shelters or veterinary clinics, consistency of personnel interacting with the dogs, predictability of handling activities, and use of low stress handling techniques can reduce stress and improve welfare. Given the considerable individual variation in the dogs' physiological and behavioral reactions to handling in this study, staff handling animals regularly need to be well-versed in reading canine body language, particularly signs of stress, and adjust their approach when necessary to reduce stress and improve welfare (69). This finding supports recommendations for optimal care of shelter dogs [e.g., (77)], and aligns with findings reported for cats housed in cages (78). In addition, a short mindfulness activity, conducted immediately prior to working with the dogs, may have had a stabilizing effect on the emotional state of the handlers, although further research is needed to confirm this. We did not find strong evidence of emotional contagion between dogs and unfamiliar handlers in this study, perhaps due to the brief duration of the relationship between human and dog in this setting.

## Data Availability Statement

The raw data supporting the conclusions of this article will be made available by the authors, without undue reservation.

## Ethics Statement

The studies involving human participants were reviewed and approved by UC Davis Institutional Research Board. The patients/participants provided their written informed consent to participate in this study. The animal study was reviewed and approved by the UC Davis Institutional Animal Care and Use Committee (IACUC). Written informed consent was obtained from the owners for the participation of their animals in this study.

## Author Contributions

EG and LH: conceived the study. EG, LH, MB, JS, JB, and AAT: developed the study design protocols for behavioral and cardiac activity data collection. DD, LH, and LK: developed the study design protocols for the mindfulness intervention and comparison. APT: conducted participant recruitments and initial screening. SL: coordinated behavioral data coding using BORIS. JS and RH: completed cardiac data processing in Kubios. EG, SL, APT, KW, and DD: collected the video and cardiac data from dogs and human handlers. EG: conducted the data analysis and drafted the initial manuscript. EG, SL, DD, KW, MB, JS, RH, LK, JB, AAT, APT, and LH: reviewed and revised the final manuscript for submission. All authors contributed to the article and approved the submitted version.

## Funding

Funding for this study was provided by the Koret Shelter Medicine Program, UC Davis Center for Companion Animal Health (CCAH), School of Veterinary Medicine, Grant#: 2018-64-KSMP.

## Conflict of Interest

DD owns company StressReductionPrograms.com. The remaining authors declare that the research was conducted in the absence of any commercial or financial relationships that could be construed as a potential conflict of interest.

## Publisher's Note

All claims expressed in this article are solely those of the authors and do not necessarily represent those of their affiliated organizations, or those of the publisher, the editors and the reviewers. Any product that may be evaluated in this article, or claim that may be made by its manufacturer, is not guaranteed or endorsed by the publisher.
