# Supplementary material for: Assessing the Relationship Between Emotional States of Dogs and Their Human Handlers, Using Simultaneous Behavioral and Cardiac Measures
Source: Front Vet Sci. 2022 Jul 11;9:897287. doi: 10.3389/fvets.2022.897287 (PMC9310693; doi:10.3389/fvets.2022.897287)
Supplement: Supplementary file 1 [file Data_Sheet_1.docx]

 Manuscript 897287-Revision 1 Grigg et al. Assessing the relationship between emotional states of dogs and their human handlers, using simultaneous behavioral and cardiac measures

**Supplementary Material:**

**S1:** Results of the repeated measures ANOVA analyses on canine cardiac activity parameters (difference from baseline, BL) by handling set#, session (1 or 2), and pre-handling activity (mindfulness vs. control): A) mean HR, B) RMSSD, C) HF (log).

**A) mean HR (canine):**

| **Test for between subject effects:** | |  | |  | |  | |  | |  |
| --- | --- | --- | --- | --- | --- | --- | --- | --- | --- | --- |
| **Source** | **DF** | **Sum of squares** | | **Mean squares** | | **F** | | **Pr > F** | |  |
| Session | 1 | 851.157 | | 851.157 | | 0.823 | | 0.369 | |  |
| Mindfulness/Control | 1 | 0.948 | | 0.948 | | 0.001 | | 0.976 | |  |
| Session*Mindfulness/Control | 1 | 1039.327 | | 1039.327 | | 1.005 | | 0.321 | |  |
| Error | 50 | 51700.986 | | 1034.020 | |  | |  | |  |
|  |  | |  | |  | |  | |  | |
| **Test for within subject effects:** |  | |  | |  | |  | |  | |
| **Source** | **DF** | | **Sum of squares** | | **Mean squares** | | **F** | | **Pr > F** | |
| Repetition | 2 | | 2282.284 | | 1141.142 | | 4.203 | | **0.017** | |
| Session*Repetition | 2 | | 37.758 | | 18.879 | | 0.070 | | 0.933 | |
| Mindfulness/Control*Repetition | 2 | | 207.529 | | 103.764 | | 0.382 | | 0.683 | |
| Session*Mindfulness/Control*Repetition | 2 | | 454.651 | | 227.326 | | 0.837 | | 0.435 | |
| Error | 101 | | 27423.268 | | 271.518 | |  | |  | |

**B) RMSSD (canine):**

| **Test for between subject effects:** | |  |  |  |  |
| --- | --- | --- | --- | --- | --- |
| **Source** | **DF** | **Sum of squares** | **Mean squares** | **F** | **Pr > F** |
| Session | 1 | 9875.028 | 9875.028 | 2.322 | 0.134 |
| Mindfulness/Control | 1 | 447.627 | 447.627 | 0.105 | 0.747 |
| Session*Mindfulness/Control | 1 | 7965.844 | 7965.844 | 1.873 | 0.178 |
| Error | 47 | 199856.308 | 4252.262 |  |  |

| **Test for within subject effects:** | |  |  |  |  |
| --- | --- | --- | --- | --- | --- |
| **Source** | **DF** | **Sum of squares** | **Mean squares** | **F** | **Pr > F** |
| Repetition | 2 | 5200.136 | 2600.068 | 3.219 | **0.043** |
| Session*Repetition | 2 | 1395.144 | 697.572 | 0.864 | 0.424 |
| Mindfulness/Control*Repetition | 2 | 974.824 | 487.412 | 0.604 | 0.548 |
| Session*Mindfulness/Control*Repetition | 2 | 3159.878 | 1579.939 | 1.956 | 0.145 |
| Error | 95 | 76722.049 | 807.601 |  |  |

**C) HF (log) (canine):**

| **Test for between subject effects:** |  |  |  |  |  |
| --- | --- | --- | --- | --- | --- |
| **Source** | **DF** | **Sum of squares** | **Mean squares** | **F** | **Pr > F** |
| Session | 1 | 60.459 | 60.459 | 1.327 | 0.255 |
| Mindfulness/Control | 1 | 49.554 | 49.554 | 1.088 | 0.302 |
| Session*Mindfulness/Control | 1 | 68.237 | 68.237 | 1.498 | 0.227 |
| Error | 48 | 2186.770 | 45.558 |  |  |

| **Test for within subject effects:** |  |  |  |  |  |
| --- | --- | --- | --- | --- | --- |
| **Source** | **DF** | **Sum of squares** | **Mean squares** | **F** | **Pr > F** |
| Repetition | 2 | 15.974 | 7.987 | 1.197 | 0.305 |
| Session*Repetition | 2 | 10.677 | 5.338 | 0.800 | 0.451 |
| Mindfulness/Control*Repetition | 2 | 3.585 | 1.793 | 0.269 | 0.765 |
| Session*Mindfulness/Control*Repetition | 2 | 37.165 | 18.582 | 2.785 | 0.065 |
| Error | 97 | 647.101 | 6.671 |  |  |

**S2:** Results of the repeated measures ANOVA analyses on human cardiac activity parameters (difference from baseline, BL) by handling set#, session (1 or 2), and pre-handling activity (mindfulness vs. control): A) mean HR, B) RMSSD, C) HF (log).

**A) mean HR (human):**

| **Test for between subject effects:** | | |  |  |  |
| --- | --- | --- | --- | --- | --- |
| **Source** | **DF** | **Sum of squares** | **Mean squares** | **F** | **Pr > F** |
| Session | 1 | 10.341 | 10.341 | 0.023 | 0.880 |
| Mindfulness/Control | 1 | 10.568 | 10.568 | 0.023 | 0.879 |
| Session*Mindfulness/Control | 1 | 44.597 | 44.597 | 0.099 | 0.755 |
| Error | 47 | 21245.936 | 452.041 |  |  |

| **Test for within subject effects:** | | |  |  |  |
| --- | --- | --- | --- | --- | --- |
| **Source** | **DF** | **Sum of squares** | **Mean squares** | **F** | **Pr > F** |
| Repetition | 2 | 264.876 | 132.438 | 2.337 | 0.100 |
| Session*Repetition | 2 | 19.940 | 9.970 | 0.176 | 0.839 |
| Mindfulness/Control*Repetition | 2 | 55.443 | 27.722 | 0.489 | 0.614 |
| Session*Mindfulness/Control*Repetition | 2 | 152.709 | 76.355 | 1.348 | 0.263 |
| Error | 95 | 5382.660 | 56.660 |  |  |

**B) RMSSD (human):**

| **Test for between subject effects:** | | |  |  |  |
| --- | --- | --- | --- | --- | --- |
| **Source** | **DF** | **Sum of squares** | **Mean squares** | **F** | **Pr > F** |
| Session | 1 | 2113.255 | 2113.255 | 0.859 | 0.359 |
| Mindfulness/Control | 1 | 79.019 | 79.019 | 0.032 | 0.859 |
| Session*Mindfulness/Control | 1 | 642.170 | 642.170 | 0.261 | 0.612 |
| Error | 47 | 115619.834 | 2459.996 |  |  |

| **Test for within subject effects:** | | |  |  |  |
| --- | --- | --- | --- | --- | --- |
| **Source** | **DF** | **Sum of squares** | **Mean squares** | **F** | **Pr > F** |
| Repetition | 2 | 53.462 | 26.731 | 0.440 | 0.645 |
| Session*Repetition | 2 | 4.181 | 2.090 | 0.034 | 0.966 |
| Mindfulness/Control*Repetition | 2 | 80.248 | 40.124 | 0.660 | 0.518 |
| Session*Mindfulness/Control*Repetition | 2 | 389.217 | 194.609 | 3.202 | **0.044** |
| Error | 95 | 5774.620 | 60.785 |  |  |

**C) HF (log) (human):**

| **Test for between subject effects:** |  |  |  |  |  |
| --- | --- | --- | --- | --- | --- |
| **Source** | **DF** | **Sum of squares** | **Mean squares** | **F** | **Pr > F** |
| Session | 1 | 0.267 | 0.267 | 0.012 | 0.913 |
| Mindfulness/Control | 1 | 0.652 | 0.652 | 0.029 | 0.865 |
| Session*Mindfulness/Control | 1 | 2.237 | 2.237 | 0.101 | 0.752 |
| Error | 47 | 1042.079 | 22.172 |  |  |

| **Test for within subject effects:** |  |  |  |  |  |
| --- | --- | --- | --- | --- | --- |
| **Source** | **DF** | **Sum of squares** | **Mean squares** | **F** | **Pr > F** |
| Repetition | 2 | 3.070 | 1.535 | 0.448 | 0.640 |
| Session*Repetition | 2 | 1.836 | 0.918 | 0.268 | 0.765 |
| Mindfulness/Control*Repetition | 2 | 13.358 | 6.679 | 1.951 | 0.146 |
| Session*Mindfulness/Control*Repetition | 2 | 0.716 | 0.358 | 0.105 | 0.901 |
| Error | 95 | 325.210 | 3.423 |  |  |
